# Fuling production areas in China: climate and distribution changes (A.D. 618–2100)

**DOI:** 10.3389/fpls.2024.1289485

**Published:** 2024-01-26

**Authors:** Yunlu Jiang, Aoyu Ren, Xue Sun, Bin Yang, Huasheng Peng, Luqi Huang

**Affiliations:** ^1^ State Key Laboratory for Quality Ensurance and Sustainable Use of Dao-di Herbs, National Resource Center for Chinese Materia Medica, China Academy of Chinese Medical Sciences, Beijing, China; ^2^ Key Scientific Research Base of Traditional Chinese Medicine Heritage (Institute of Chinese Materia Medica, China Academy of Chinese Medical Sciences) State Administration of Cultural Heritage, Beijing, China; ^3^ Institute of Chinese Materia Medica, China Academy of Chinese Medical Sciences, Beijing, China; ^4^ Research Unit of DAO-DI Herbs, Chinese Academy of Medical Sciences (2019RU57), Beijing, China

**Keywords:** Fuling, *Pachyma hoelen*, MAXENT model, local chronicles, traditional Chinese medicine (TCM), *Poria cocos*, Wolfiporia

## Abstract

Through a meticulous analysis of ancient Chinese literature, this study comprehensively documents the geographical distribution of Fuling, a traditional Chinese medicinal material, during the Tang, Song, Ming, and Qing dynasties spanning from the seventh to the twentieth century in China. Based on the contemporary distribution information of Fuling, we utilized the maximum entropy (MaxEnt) model to simulate the suitable distribution areas of Fuling under both present-day conditions and in the future (2081~2100). The findings reveal that climate change has influenced the distribution of Fuling production areas. The shifts in Fuling’s origin during different periods in ancient and modern times align with climate fluctuations and concurrent societal development. During the Tang and Song dynasties, Fuling primarily originated in northern China. However, it migrated southward during the Little Ice Age (LIA) and has recently shown a slight northward shift, in line with the climate fluctuations of the LIA and contemporary global warming trends. This study offers a comprehensive analysis of the changes in the distribution and production areas of Fuling over a 1500-year period, encompassing ancient, modern, and future periods. The results provide critical insights for adjusting Fuling cultivation areas in response to climate change and for further exploration of the mechanisms through which climate impacts the growth of Fuling.

## 1 Introduction

The distribution of plants is influenced by natural environmental factors, such as temperature, precipitation, soil composition, and other elements (Yu et al., 2019). Changes in climate throughout history have significantly impacted plant distribution in ancient China, as supported by archaeological and historical records (Wang et al., 2009). Approximately 85% of components in Traditional Chinese Medicine (TCM) are derived from plants, including fungi such as Fuling, Lingzhi (*Ganoderma lucidum* and *Ganoderma sinense*), and Zhuling (*Polyporus umbellatus*). Within TCM clinical practice, the term “dao-di herbs” pertains to region-specific herbs and, using a particular production process, are chosen over time for their superior quality, efficacy, and reputation compared to herbs grown elsewhere. Both the distribution of dao-di herbs and suitable plant growth zones are affected by climate conditions.

Fuling, a traditional Chinese medicinal material derived from the dried sclerotia of *Pachyma hoelen* Fr. (Wu et al., 2020; Papp and Dai, 2022), has been utilized for over 2000 years. Its benefits include promoting water infiltration, strengthening the spleen, and calming the heart. Fuling is also utilized in food, and features prominently in traditional Chinese foods such as Fuling cake. Notably, the 2020 edition of the Chinese Pharmacopoeia lists 239 types of Chinese patent medicines containing Fuling (Chinese Pharmacopoeia Commission, 2020). In recent years, Fuling produced in China has been exported to countries including Japan, South Korea, Vietnam, and Malaysia. From 2011 to 2020, the average annual export volume of Fuling from mainland China was 7,485.91 t, with an average annual export value of 30,376,900 US dollars (Jin et al., 2022). The development of the Fuling industry, particularly the production of high-quality Fuling, has the potential to enhance the income of individuals engaged in Fuling cultivation in mountainous regions.

Ancient Chinese literature contains extensive historical documentation of medicines. The production region of Fuling has been documented in various Chinese medical texts since the *Ming Yi Bie Lu* (*名医别录*) during the Southern and Northern Dynasties period (A.D. 420~589). Abundant historical documents depict Fuling’s distribution across different periods. Medical literature from past dynasties indicates that during the Tang and Song dynasties, Fuling widely distributed in north China, with Shandong province being its primary production region. However, in modern times, the main production regions of Fuling have shifted to the southern areas, such as the Dabieshan Mountains and the Yunnan-Guizhou region. Notably, there is no natural or cultivated presence of Fuling in Shandong Province any more. Historical geography reveals climatic variations. The Tang and Song dynasties, spanning the 7th to 13th centuries, experienced relatively warm conditions. The Ming and Qing Little Ice Age (LIA) (15th to 19th century) brought widespread cooling to China. Due to Fuling’s preference for a warm and humid environment, its distribution area is expected to undergo changes in response to variations in temperature and humidity.

Since the Tang and Song dynasties, China’s climate has changed. Investigating whether the production areas and authentic producing areas of Fuling have undergone corresponding changes can offer new insights into understanding the historical and contemporary changes in the authentic producing areas of Fuling. Additionally, in the context of global warming, some plants are anticipated to shift their distribution to adapt to the warming climate (Richard and David, 2013). Therefore, the production area of Fuling in China may potentially migrate northward and revert to its distribution position prior to the LIA. Studying the changes in the Fuling production areas under the influence of future climate can serve as a foundation for the development of the Fuling industry.

In this paper, we conducted a review of ancient texts on Fuling since the Tang Dynasty in China, and preliminarily reconstructed the historical distribution area of Fuling based on the distribution data found in historical materials. The study also introduced the MaxEnt model to aid in analyzing the impact of climate on the variation of Fuling production areas. Building upon these research methods, the paper addresses four key issues: (1) the correlation between climate and the contemporary distribution of Fuling; (2) categorizing Fuling distribution regions during the Tang, Song, Ming, and Qing dynasties based on ancient Chinese records; (3) assessing the influence of historical climate variations on Fuling distribution areas; and (4) potential changes in Fuling production areas in the context of future climate warming.

## 2 Materials and methods

### 2.1 Collecting and mapping historical resources

#### 2.1.1 Collection of historical documents

In this study, we primarily collected historical resources from the Tang (A.D. 618~907), Song (A.D. 960-1279), Ming (A.D. 1368-1644), and Qing (A.D. 1636~1912) dynasties.

The historical documents from the Tang Dynasty include *Xin Tang Shu Di Li Zhi* (*新唐书地理志*), *Jiu Tang Shu Di Li Zhi* (*旧唐书地理志*), *Tang Liu Dian* (*唐六典*), *Tong Dian* (通*典*), *Xin Xiu Ben Cao* (*新修本草*), *Yuan He Jun Xian Tu Zhi* (*元和郡县图志*), *Qian Jin Yi Fang* (*千金翼方*), and *Quan Tang Shi* (*全唐诗*). Similarly, the historical documents from the Song Dynasty include *Song Hui Yao* (*宋会要*), *Song Shi Di Li Zhi* (*宋史地理志*), *Yuan Feng Jiu Yu Zhi* (*元丰九域志*), *Ben Cao Tu Jing* (*本草图经*), *Tai Ping Huan Yu Ji* (*太平寰宇记*), *Tai Ping Yu Lan* (*太平御览*), *Shao Xing Ben Cao* (*绍兴本草*), *Quan Song Shi* (*全宋诗*), *Dong Yuan Lu* (*东原录*), *Meng Liang Lu* (*梦粱录*), and local chronicles of the Song Dynasty.

The historical documents from the Ming and Qing dynasties are primarily comprised of local chronicles, which can be accessed on the website of Erudition-China Fangzhi Library (http://dh.ersjk.com/spring/user/mlogin) and National Library of China-National Digital Library of China (http://www.nlc.cn/)(**Supplementary Figure 1**). In this study, a total of 676 local chronicles from the aforementioned three dynasties were examined to extract valuable information regarding the origin of Fuling. The relevant records pertaining to Fuling were consolidated and categorized under the headings of “*Wu Chan* (*物产*)”, “*Tu Chan* (*土产*)” and “*Tu Gong* (*土贡*)” within these local chronicles.

#### 2.1.2 Mapping Fuling distribution

To visually represent the distribution area of Fuling in different historical periods, we transformed the geographical locations mentioned in ancient books into modern locations and created distribution area maps of Fuling for the four dynasties based on the modern map of China. We utilized ArcMAP 10.8 for data processing, employing the GCS_WGS_1984 geographical coordinate system. The map data was obtained from the natural resources of the People’s Republic of China (http://bzdt.ch.mnr.gov.cn/), which provides a 1:11,000,000 map of China and an administrative zoning map of China. According to the ancient local chronicles, we hypothesized that the frequency of Fuling’s mention in these chronicles correlates with its local production. Therefore, we counted the number of times Fuling was recorded in the local chronicles of each period to estimate the amount of Fuling holdings.

### 2.2 MaxEnt model

#### 2.2.1 Species occurrence data

For this study, we collected distribution data for Fuling from ten provinces: Anhui, Yunnan, Hubei, Hunan, Jiangxi, Guizhou, Henan, Fujian, Shaanxi, and Guangxi. To enhance the comprehensiveness of the present distribution data, we supplemented it with information from relevant literature (Sun, 2016).

To avoid overfitting due to close proximity between recording points, we utilized the ENMTools software to remove duplicate recording data within the 1km × 1km grid cells (Warren et al., 2009). Ultimately, we obtained distribution information for 84 current Fuling samples (**Supplementary Figure 2**).

#### 2.2.2 Screening of environmental variables

To simulate the distribution of suitable locations for Fuling, this study utilized 19 environmental variables, including temperature and precipitation. The current climate data (1970~2000) and the future climate data (2081~2100) were obtained from the WorldClim Data website (https://worldclim.org/), with spatial resolutions of 30 arc sec. Data for the future period are obtained from the sixth phase of the International Coupled Model Comparison Program (CMIP6) using the medium Resolution Climate System Model (BCC-CSM2-MR) released by the second Generation National (Beijing) Climate Center. We select three shared socio-economic pathways (SSPs) from the IPCC 6th Emissions Report (Zhang et al., 2020): sustainable development (SSP1-2.6), localized development (SSP3-7.0), and conventional development (SSP5-8.5). In order to mitigate overfitting caused by multicollinearity among the variables, a preliminary experiment was conducted. The significance and contribution rate of the environmental variables were tested using the jackknife method, and Pearson correlation coefficients between the distribution of Fuling and the environmental variables were calculated using SPSS. If the Pearson correlation coefficient exceeded 0.85, the variable with a lower contribution was removed, in accordance with the results of the jackknife test (Bian and Shi, 2019). Ultimately, eight key environmental factors were retained to simulate the distribution of suitable areas for Fuling (**Table 1**).

**Table 1:** Leading environmental variables selected in the model.

Code	Environmental variables	Units
bio3	Isothermality	-
bio6	Min Temperature of Coldest Month	°C
bio8	Mean Temperature of Wettest Quarter	°C
bio10	Mean Temperature of Warmest Quarter	°C
bio13	Precipitation of Wettest Month	mm
bio14	Precipitation of Driest Month	mm
bio16	Precipitation of Wettest Quarter	mm
bio18	Precipitation of Warmest Quarter	mm

#### 2.2.3 Establishment of MaxEnt model and parameter setting

The MaxEnt software (version 3.4.3) was employed to import the distribution data (.csv) and the set of environmental variables (.asc). The following parameters were configured: jackknife analysis was used to assess the significance and contribution rate of the environmental variables; a forecast distribution diagram and an environmental response curve were generated; 25% of the occurrence data were randomly selected for testing; the output format was logistic, and the remaining parameters were set to their default values (Lan et al., 2022; Zhou et al., 2022). The modeling process was repeated 10 times, and the average of these repetitions was included in the output result file. Furthermore, the area under the curve (AUC) of the receiver operating characteristics (ROC) was used to evaluate the reliability of the model’s predictions. It is generally accepted that an AUC value greater than 0.9 indicates highly accurate predictions that can be relied upon.

#### 2.2.4 Processing of prediction results

Using ArcMap, distribution maps of Fuling-suitable locations were created by overlaying the results obtained from the MaxEnt model computations. The distribution area of Fuling was categorized into four levels using the natural breaks (Jenks) approach. The level of suitability for Fuling in each region was estimated based on the distribution probability (*P*): uninhabitable area (*P* < 0.25), low suitable area (0.25 ≤*P* < 0.5), medium suitable area (0.5 ≤ *P* < 0.7), high suitable area (*P* ≥ 0.7).

## 3 Results

### 3.1 Distribution area of Fuling in Tang and Song dynasties

China’s Tang Dynasty, which lasted from A.D. 618 to 907, was followed by the subsequent Song Dynasty (A.D. 960-1279), which was further divided into the Northern Song and Southern Song, with A.D. 1127 serving as the dividing line. The temperature was higher in the Tang and Song eras than in the Ming and Qing dynasties. China’s climate has shifted from warm to cold during the last 8,000 years. Tang Dynasty until the middle of the Northern Song Dynasty (A.D. 1050) represented a relatively warm phase, with the distribution of citrus and lychee extending further north than today (Wen, 2019). Subsequently, from the middle of the Northern Song Dynasty until the middle of the Ming Dynasty, China encountered a cooler period, still surpassing the LIA in temperature.

#### 3.1.1 Distribution of Fuling production areas during the Tang Dynasty

**Figure 1:**
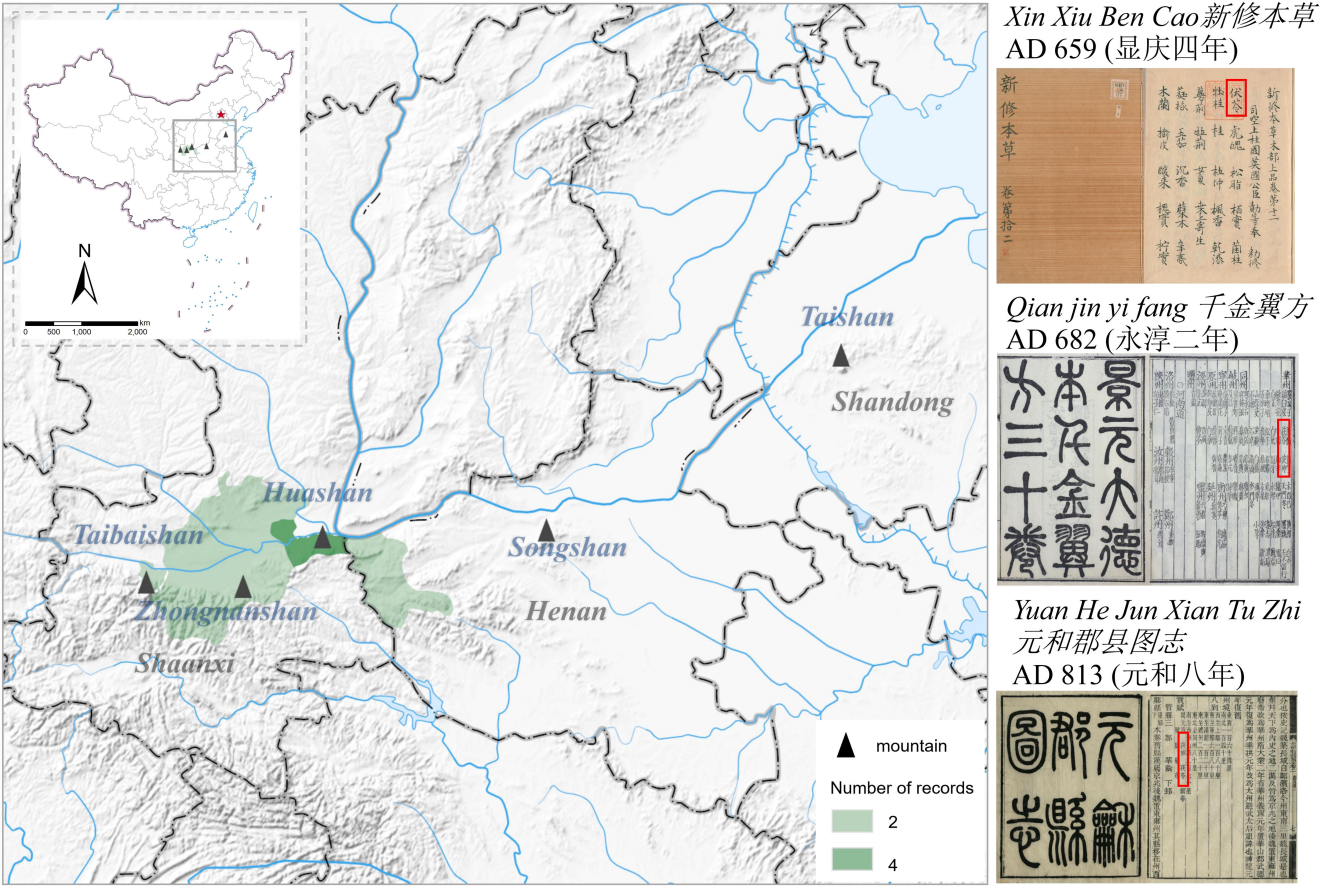
Distribution of Fuling production areas during the Tang Dynasty.

The distribution map of Fuling during the Tang Dynasty is presented in this article (**Figure 1**), utilizing historical data. The northern region comprises Shaanxi, Shandong, and Henan provinces, which constituted the primary distribution zones for Fuling during the Tang Dynasty. A total of five ancient books from the Tang Dynasty recorded that Fuling was predominantly produced in Huazhou Prefecture (华州), Shaanxi Province. The locally produced Fuling exhibited high quality and was presented as tribute to the emperor during the Tang Dynasty.

Yongzhou Prefecture (雍州), also in Shaanxi Province, contributed to Fuling production, as documented in *Qian jin Yi Fang* (*千金翼方*). According to *Xin xiu ben cao* (*新修本草*) records, Huazhou Prefecture yielded the highest-quality Fuling, followed by Yongzhou Prefecture. Fuling was also present in Taishan (泰山), a mountain in Shandong Province, although the quality was not on par with that of Shaanxi Province. Another Fuling production area, as reported in *Qian jin Yi Fang*, was Guozhou Prefecture (虢州), situated at the boundary between Henan and Shaanxi provinces.

In ancient China, it was believed that Fuling was formed by condensing the essence of pine trees and grew under old pines in high mountains. Therefore, the ancient books of the Tang Dynasty also documented the presence of several large mountains in the production of Fuling, such as Huashan (华山), Zhongnanshan (终南山), Taibaishan (太白山) in Shaanxi Province, Songshan (嵩山) in Henan Province, and Taishan in Shandong Province. Among them, Huashan, Songshan, and Taishan are among the five most famous mountains in China, which aligns with the ancient records indicating that Fuling is produced under the large pines in the deep mountains.

#### 3.1.2 Distribution of Fuling production areas during the Song Dynasty

**Figure 2:**
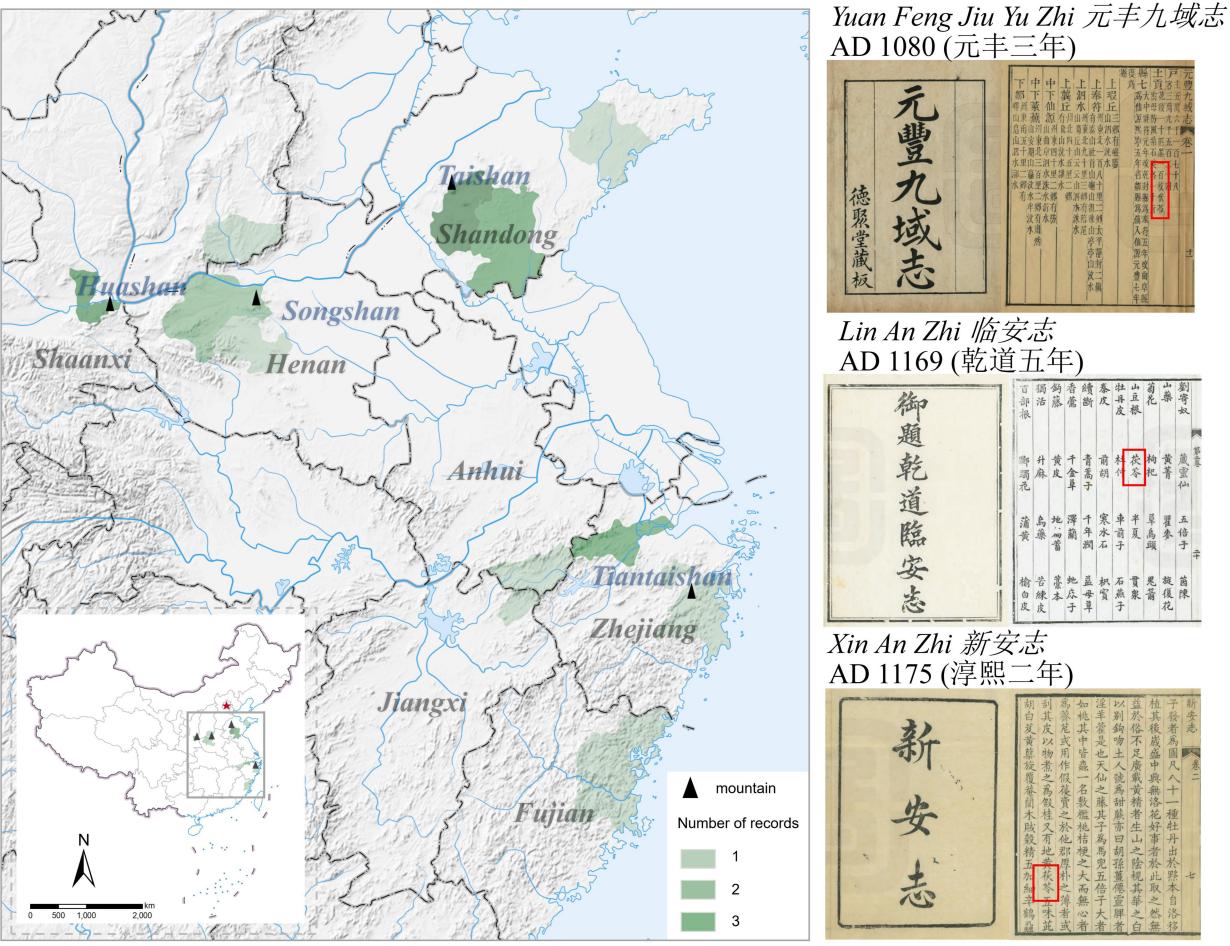
Distribution of Fuling production areas during the Song Dynasty.

The distribution map of Fuling’s cultivation during the Song Dynasty was generated from Song Dynasty literature (**Figure 2**). The distribution range of Fuling expanded during the Song Dynasty compared to that of the Tang Dynasty, encompassing Shaanxi, Henan, Shandong, and Shanxi in the north, and Anhui, Zhejiang, and Fujian provinces in the south.

Historical records like the *Song shi di li zhi* (*宋史地理志*) indicate that Yizhou Prefecture (沂州) and Yanzhou Prefecture (兖州) of Shandong Province, as well as Huazhou Prefecture of Shaanxi Province, all contributed tribute to the imperial court, signifying the primary production regions of Fuling were assigned to Shandong and Shaanxi provinces. The distribution records of Fuling in the south during the Song Dynasty are all sourced from the ancient books of the Southern Song Dynasty. At that time, the capital of Song Dynasty was relocated to Lin’an Prefecture (临安), Zhejiang Province. Notably, *Qian Dao Lin An Zhi* (*乾道临安志*), *Xian Chun Lin An Zhi* (*咸淳临安志*) and *Meng Liang Lu* (*梦粱录*) all documented the production of Fuling in Lin’an Prefecture. Additionally, Xin’an Prefecture (新安) in Anhui Province and Fuzhou Prefecture (福州) in Fujian Province, both in southern China, also recorded the production of Fuling.

### 3.2 Distribution of Fuling production areas during the Ming and Qing dynasties

The Ming Dynasty, which lasted from A.D. 1368 to 1644, and the Qing Dynasty, covering A.D. 1636 to 1912, coincided with a relatively cold period known as the LIA that occurred globally between the early 15th and early 20th centuries (Matthes, 1939). China experienced its coldest period in 5,000 years during the mid-16th to mid-19th century, which aligned with the Ming and Qing dynasties, leading to the designation of the Ming and Qing LIA (Zhu, 1973).

#### 3.2.1 Distribution area of Fuling production areas in the Ming Dynasty

**Figure 3:**
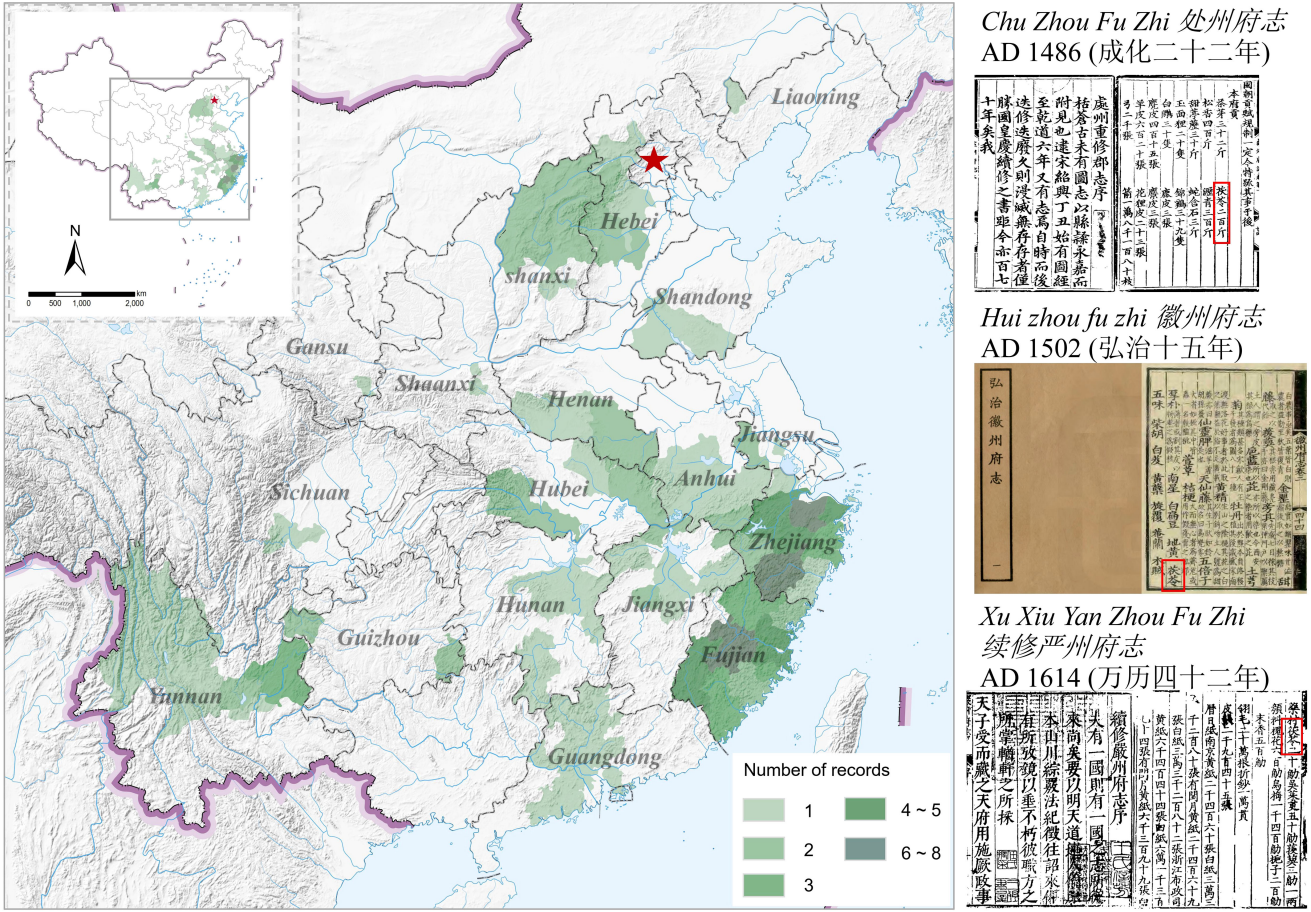
Distribution of Fuling production areas during the Ming Dynasty.

A total of 144 pieces of information on Fuling’s origin were compiled in the local chronicles of the Ming Dynasty (**Figure 3**). The overall distribution of Fuling in the Ming Dynasty was extensive, encompassing 18 provinces including Zhejiang, Fujian, Anhui, Yunnan, Hunan, and Hubei. Within the Ming Dynasty, the distribution of Fuling is more frequently documented in Zhejiang and Fujian Provinces. Specifically, there were 6 recorded instances in Chuzhou Prefecture (处州) and Shaoxing Prefecture (绍兴) in Zhejiang Province, and 8 recorded instances in Yanping Prefecture (延平), Fujian Province. Fuling was widely distributed in these two provinces, indicating high production during the Qing Dynasty. The *Jia Jing Ning Guo Xian Zhi* (*嘉靖宁国县志*) (A.D. 1549) documented that Ningguo County, under the jurisdiction of Ningguo Prefecture in Anhui Province, paid tribute to the emperor with Fuling. Similarly, the *Guang Xu Fen Shui Xian Zhi* (*光绪分水县志*) (A.D. 1907) recorded that Fenshui County in Zhejiang Province paid tribute to the court with Fuling during the Ming Dynasty. Ningguo County (宁国) is located in southeastern Anhui Province, while Fenshui County (分水) is located in northwestern Zhejiang Province, indicating their geographical adjacency. Local chronicles from the Ming Dynasty suggest that Anhui Province and Zhejiang Province were the authentic producing areas of Fuling, and the quality of Fuling produced at the junction of the two provinces was high. Compared with the Tang and Song dynasties, the distribution area of Fuling in the Ming Dynasty underwent significant changes. Both the main producing areas and the authentic producing areas shifted from the north to the south, and the distribution range expanded significantly.

#### 3.2.2 Distribution of Fuling production areas in the Qing Dynasty

**Figure 4:**
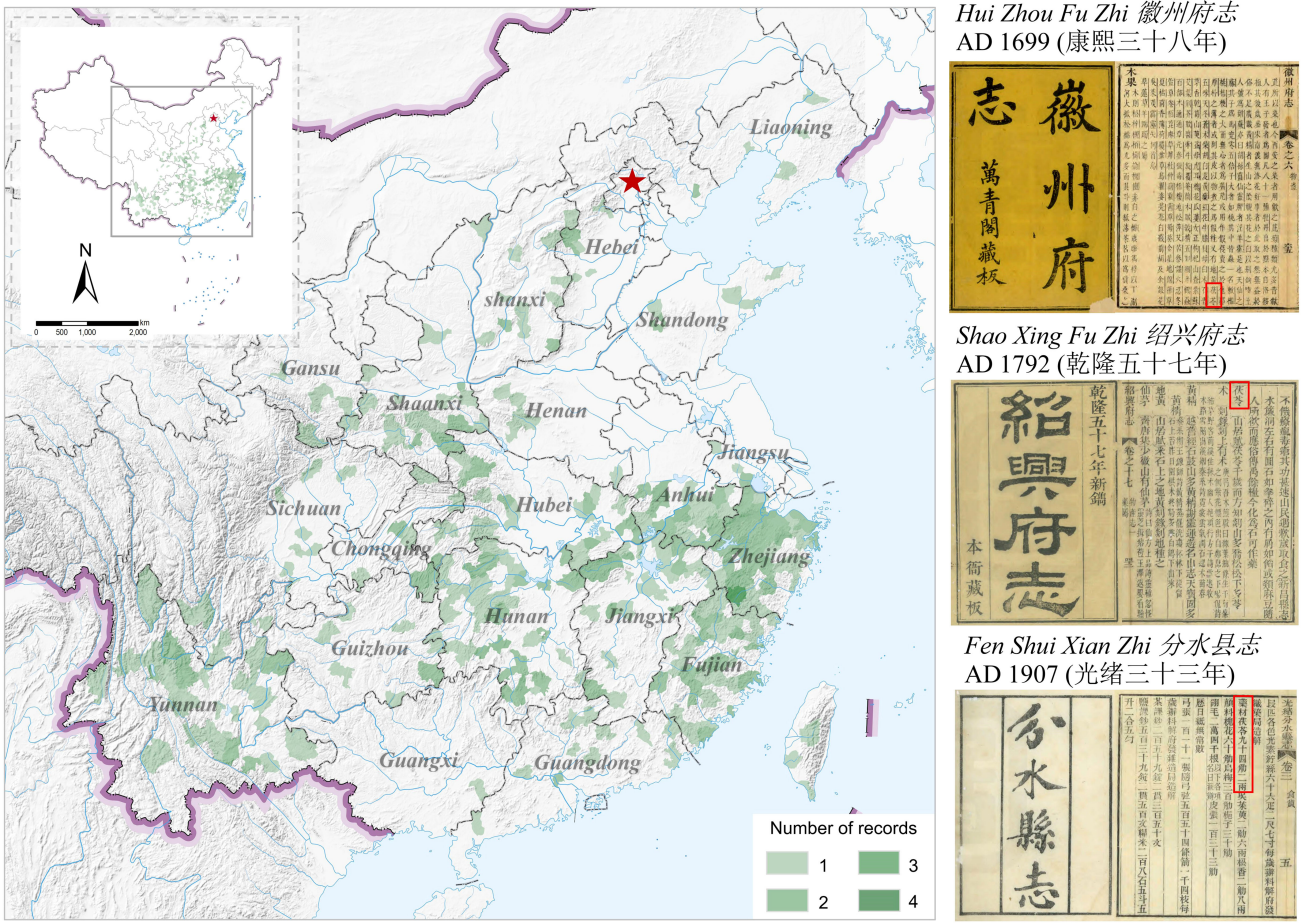
Distribution of Fuling production areas during the Qing Dynasty.

The Qing Dynasty, which closely approaches modern times, stands as the final feudal dynasty in Chinese history. A plethora of local chronicles from the Qing Dynasty has been meticulously preserved. This study methodically organizes 588 local records detailing Fuling’s production area during the Qing Dynasty, culminating in a regional map (**Figure 4**) depicting Fuling’s Qing Dynasty production expanse. Due to the more accurate geographical information in the Qing Dynasty’s local chronicles relative to other eras, the resultant map achieves a county-level precision, effectively portraying the Qing Dynasty’s Fuling distribution.

Fuling was distributed across 23 provinces in the Qing Dynasty, including Anhui, Yunnan, Zhejiang, and Fujian. According to the local chronicles of the Qing Dynasty, Yunnan, Anhui, and Zhejiang Province were the primary producing areas of Fuling during that period, with Yunnan and Anhui being the authentic producing areas of Fuling. Fuling was distributed extensively in Yunnan Province. During the Qing Dynasty, Yunnan Province governed a total of 23 prefectures, with 18 areas producing Fuling, as documented in local Chronicles. Numerous local chronicles and ancient books in Yunnan indicated that the quality of Yunnan Fuling is the best in the country, such as the *Xuan Tong Meng Zi Xian Zhi* (*宣统蒙自县志*) (1911), which stated that Yunling is renowned worldwide. Yunling is the Fuling produced in Yunnan Province. Fuling was exclusively produced in the southern part of Anhui Province, with Anqing Prefecture (安庆) in southwestern Anhui being the primary producing area of Fuling during the Qing Dynasty. The distribution range of Fuling in the Qing Dynasty was largely consistent with that of the Ming Dynasty, with the main producing areas migrating to the west and northwest. This shift resulted in a change from Zhejiang Province and Yunnan Province to Yunnan Province and the Dabieshan Mountains in Anhui Province, among other locations.

### 3.3 The contemporary distribution areas of Fuling and its key climatic factors

**Figure 5:**
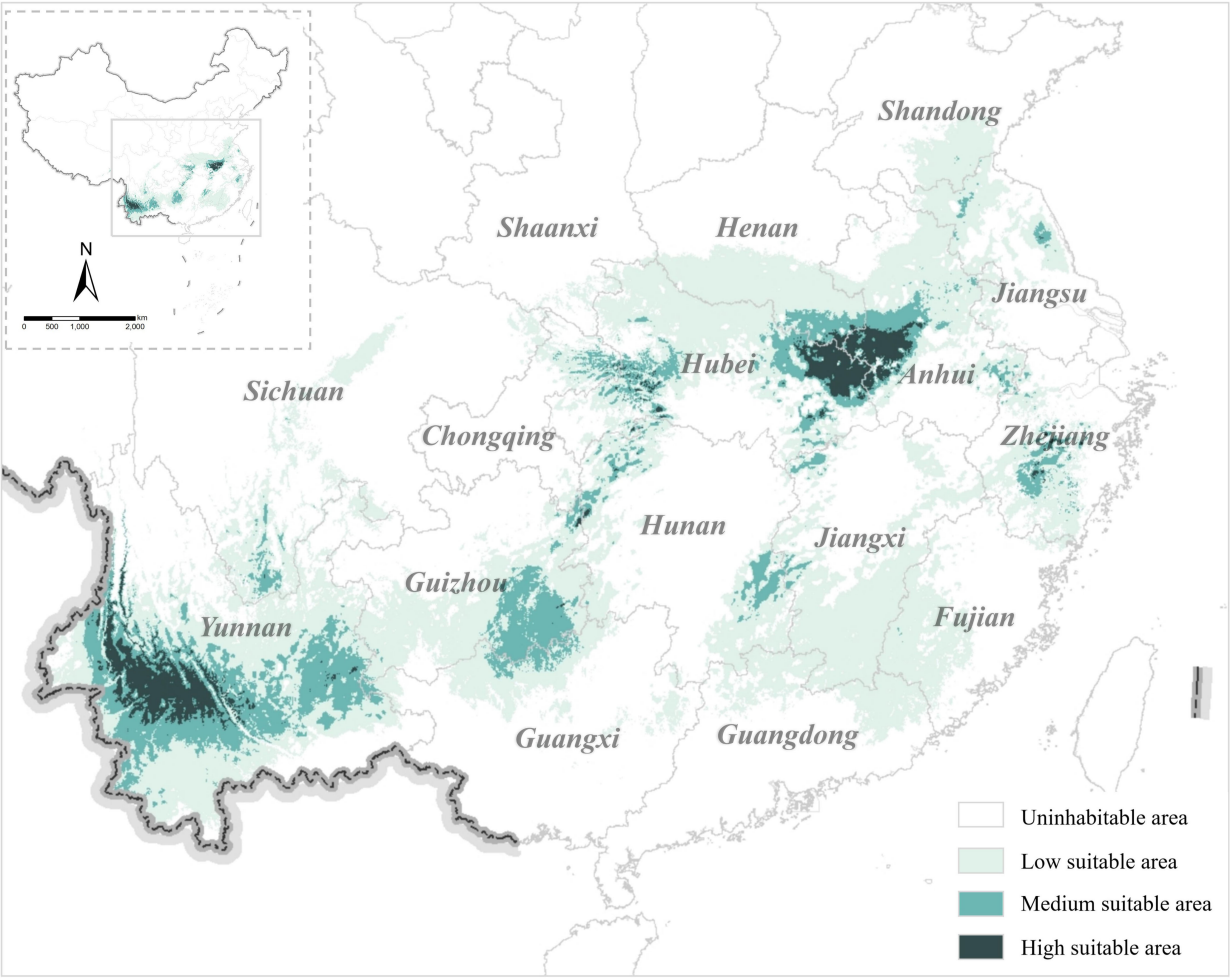
Fuling distribution suitable area in modern times.

The model’s prediction performance was evaluated using the average AUC value obtained from 10 cross-validation operations. The average AUC value of the modern Fuling distribution model is 0.954 (**Supplementary Figure 3**). A value exceeding 0.9 indicates accurate model performance, enabling simulation of the Fuling distribution area. The MaxEnt model-simulated suitable distribution area for modern Fuling (P > 0.2) aligned with the current Fuling distribution. The proper Fuling production area encompasses the Dabieshan Mountains and the central part of Yunnan Province, including Pu’er City (普洱) and Lincang City (临沧) in Yunnan Province, Jinzhai County (金寨), Huoshan County (霍山), and Yuexi County (岳西) in Anhui, Macheng City (麻城), Luotian County (罗田), Yingshan County (英山), and Qichun County (蕲春) in Hubei Province, and Shangcheng County (商城) and Gushi County (固始) in Henan Province (**Figure 5**). Medium and low suitable areas are primarily in provinces like Hunan, Zhejiang, Jiangxi, and Guizhou, which remain major Fuling cultivation sites today.

The distribution of modern Fuling is influenced by key climate factors, selected through the utilization of climate factor contribution rates and the Jackknife technique. The climatic factors affecting the distribution of Fuling in descending order of importance are bio14, bio6, bio3, bio16, bio8, bio10, bio13, bio18. Notably, the top four climate factors collectively account for 91.4% of the total contribution. Based on the species response curve simulated by MaxEnt model, this paper will analyze the distribution of suitable areas and limiting factors of Fuling in modern times. Fuling thrives under specific climatic conditions that bio14 exceeded 6.67 mm, bio6 ranged from -19.53 to 3.94°C, bio3 exceeded 26.68°C, and bio16 exceeded 442.23 mm.

### 3.4 Fuling distribution areas and the main climatic factors in the future from 2081 to 2100

The average AUC values of the Fuling distribution models under the three scenarios of SSP126, SSP370, and SSP585 in the 2090s were 0.954, 0.954, and 0.953, respectively, all exceeding 0.9 (**Supplementary Figure 3**). They can be utilized to simulate the distribution of Fuling in the suitable area. The area of the suitable zone under SSP126 in the future period is 9.26 × 10^5^ km^2^, with the high suitable area still mainly distributed in Yunnan and the Dabieshan Mountains, albeit with a reduced distribution area. Medium suitable areas are distributed in Yunnan, Guizhou, Anhui, Hubei, Jiangsu, Zhejiang, and other provinces. Low suitable areas are distributed in Yunnan, Guizhou, Guangxi, Jiangxi, Hunan, Fujian, Anhui, Henan, Hubei, Zhejiang, Jiangsu, Shandong, etc. The area of suitable zones under SSP370 is 9.16 × 10^5^ km^2^, with the area near the border of Guizhou and Guangxi, and part of Hunan Province transforming from medium suitable areas to low suitable areas, and part of the low suitable areas in Fujian and Zhejiang provinces transforming to medium suitable areas. The area of suitable areas under SSP585 is 9.19 × 10^5^ km^2^, with some medium suitable areas in western Hubei Province converting to low suitable areas, while some low suitable areas in Jiangsu Province are converting to medium suitable areas.

**Figure 6:**
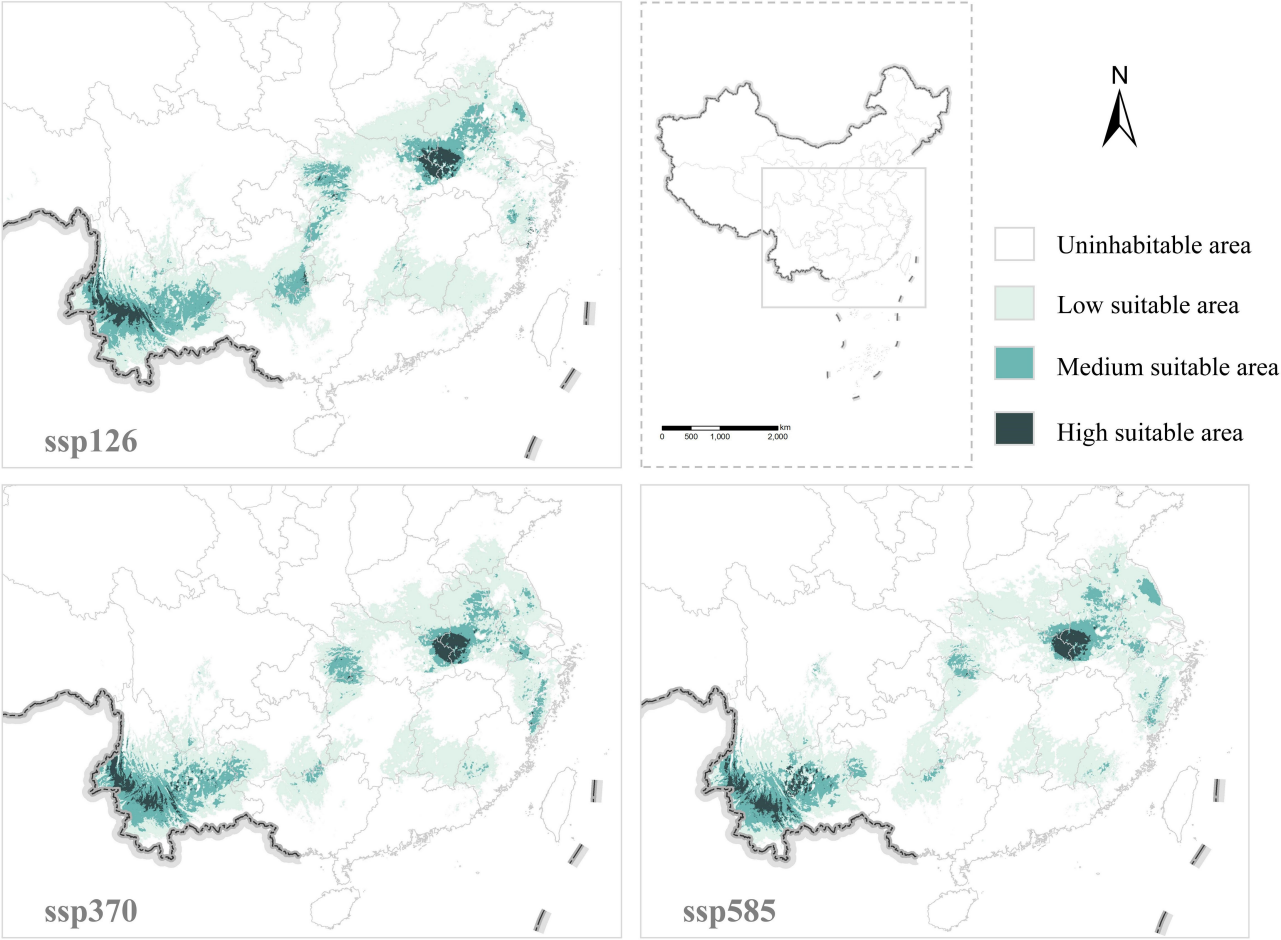
Prediction results for Fuling distribution area under different climate scenarios in the 2090s.

In 2090s, Fuling’s high suitability concentrates in Yunnan, Hubei, and Anhui; medium in Yunnan, Hubei, Anhui, Zhejiang, and Jiangsu; low in Yunnan, Sichuan, Chongqing, Guizhou, Guangxi, Hunan, Fujian, Jiangxi, Anhui, Hubei, Henan, Zhejiang, Jiangsu, and Shandong. The current suitable area is 1.02 × 10^6^ km^2^, decreasing in the future. Guangdong becomes unsuitable, while Henan’s suitable area increases. Overall, the distribution trend moves northward (**Figure 6**).

The top three climate factors influencing Fuling’s distribution under the three scenarios in 2090s were bio14, bio6, and bio3. Their contributions’ sums under SSP126, SSP370, and SSP585 were 87.9%, 86.5%, and 85%, respectively. Under SSP126, the suitable ranges for bio14 were > 14.35 mm, -8.95°C < bio6 < 6.72°C, and bio3 > 27.06. For SSP370, bio14 ranged > 14.34 mm, -8.32°C < bio6 < 8.47°C, and bio3 > 28.82. Finally, under SSP585, the suitable ranges were bio14 > 15.38 mm, -5.40°C < bio6 < 9.31°C, and bio3 > 29.71.

## 4 Discussion

### 4.1 Precipitation and temperature are important climatic factors affecting the distribution of modern Fuling production areas

The suitable distribution area Fuling of in modern times, as deduced from the MaxEnt model, is primarily concentrated in southern China, specifically in Yunnan, Anhui, Hubei, Henan, Hunan, Guizhou, Guangxi, and other provinces. This distribution aligns well with the current Fuling production areas. The simulation indicates that central Yunnan and the Dabieshan Mountains are the high suitable area for Fuling, in line with the existing production distribution. The Dabieshan Mountains are located at the junction of Anhui, Hubei, and Henan provinces, and are currently important areas for Fuling cultivation production. These include Jinzhai County, Huoshan County, and Yuexi County in Anhui Province, as well as Luotian County and Yingshan County in Hubei Province. The MaxEnt model’s simulation results strongly correlate with input distribution data, which can explain that augmenting Fuling distribution data in the Dabieshan Mountains improves simulations for both Fuling distribution in the Dabieshan Mountains and its surrounding areas. This forms a robust foundation for expanding Fuling cultivation and increasing yield.

Fuling thrives through parasitic or saprophytic associations with Pinaceae plants, such as *Pinus massoniana*, *Pinus tabuliformis*, and *Pinus hwangshanensis*. It prospers in warm and humid habitats, necessitating specific soil conditions, temperature, and humidity (Tian et al., 2017). The soil should possess excellent aeration, loose texture, permeability, and sandy loam composition, with a sand content ranging from 60% to 70%. Fuling’s optimal growth temperature ranges from 25 to 30°C, while the suitable area’s average annual temperature is around 18 to 24°C. The simulation highlights precipitation as the key factor affecting the Fuling growth, with bio14 and bio16 being the primary and fourth contributors to climate effects, aligning with Fuling’s moisture-dependent nature. Furthermore, the results indicate that Fuling growth is suitable when bio13 is < 299.79 mm. This finding concurs with a Jiangxi Province field experiment, illustrating that an overly humid climate negatively impacts Fuling growth.

Temperature is another environmental variable that influences the distribution of Fuling. The study results indicate that bio6 is the second most influential climate factor, with an acceptable distribution range of -19.53°C to 3.94°C. This range suggests that excessively low temperatures lead to slow growth and reduced production of Fuling. A field investigation was conducted in Jinzhai County, Anhui Province, within the Fuling growing region. The local Fuling has a growth cycle of approximately six months, during which it is cultivated from May to June. Harvesting takes place from November of the same year to January of the following year (Cheng et al., 2021). The coldest recorded month in Jinzhai County is January, with a minimum temperature of -2.0°C falling within the suitable temperature range for Fuling.

In conclusion, the distribution of Fuling is influenced by both temperature and precipitation. This study emphasizes that special attention is required for the dry and cold climates during the growing period of Fuling. Using Jinzhai as an illustration, ongoing temperature monitoring in January’s growth area and focused attention on December’s precipitation, the driest month, are recommended. Prompt implementation of measures for water replenishment and heat preservation is essential to ensure a fruitful Fuling yield.

### 4.2 The main production area of Fuling in northern China during the tang and song dynasties caused by warm climate

Several recent paleoclimate investigations have indicated that the Tang Dynasty coincided with a relatively warm climate. The climate during the Tang Dynasty is believed to have been warmer than today, as evidenced by the northward shift of the *Citrus reticulata* planting boundary (0.63-0.91 degrees latitude) (Wu, 2012). The results of this study demonstrate a significant change in the primary distribution area of Fuling from the Tang Dynasty to the present. During the Tang and Song dynasties, the distribution range of Fuling was limited, predominantly found in the northern regions of Shandong, Henan, and Shaanxi provinces. Presently, the primary production area of Fuling is located in the southern region of China, primarily in Yunnan, Anhui, Hubei, and other provinces. The main production area of Fuling during the Tang Dynasty was situated further north than it is today, implying that the climate during the Tang and Song dynasties was warmer than it is today.

Literature and historical data of the Tang Dynasty showed that Fuling was produced in Shaanxi Province in northern China, reflecting the warmer climate of that time, which was conducive to the growth of Fuling. The weather in the early Song Dynasty resembled the present conditions (Zhu, 1973). A cooling trend in the climate persisted from the middle of the Northern Song Dynasty until the end of the Southern Song Dynasty. During the Southern Song Dynasty, the method of cultivating Fuling was established, as stated in *Gui xin za shi* (*癸辛杂识*)(Dong et al., 2021). The capital city of the Southern Song Dynasty was relocated from Kaifeng Prefecture (开封) in northern Henan Province to Lin’an Prefecture in the south, known today as Hangzhou City in Zhejiang Province. Consequently, the introduction and cultivation of Fuling in Lin’an and its neighboring regions during this period can be attributed to the alignment with the prevailing climate and social environment of that era.

### 4.3 Fuling migration southward during Ming and Qing dynasties linked to LIA

During both the Ming and Qing dynasties, the cold LIA was encountered. The LIA of these dynasties spanned the 15th to 19th centuries, exhibiting three cold and three warm periods, peaking in 1620-1670 and 1820-1870 (Zhang, 1991). The LIA caused a national climate characterized by cold and dry conditions due to temperature cooling. This led to the prevalence of Fuling primarily in warm and humid southern and coastal areas in southeast area like Anhui, Yunnan, Zhejiang, Hunan, and others. Historical records point to Zhejiang, Yunnan, and Guizhou provinces as the key Fuling producing regions during the Ming Dynasty. In the Qing Dynasty, this shifted to Yunnan Province, Guizhou Province, and the Dabieshan Mountains, essentially in line with the current distribution.

It is documented in ancient texts that the authentic production regions of Fuling during the Ming Dynasty were Zhejiang Province, Yunnan Province, and Guizhou Province. The overall climate of the Ming Dynasty was characterized by cold temperatures, with Zhejiang, Yunnan, and Guizhou provinces were situated in the relatively warm southern region of China, conducive to the growth of Fuling. The historical records of Zhejiang Province’s tribute to the imperial court indicate the high quality of locally produced Fuling. Fuling production was documented in the southern provinces of Hunan, Jiangxi, Sichuan, Jiangsu, and Guangdong. However, in the 20th century, warming conditions led to diminished Fuling growth in Jiangsu and Guangdong provinces, aligning with the cold Ming Dynasty climate.

The LIA left a lasting impact on the climate in China, maintaining its chill into the Qing Dynasty. Fuling predominantly thrived in China’s warm and humid eastern and southern regions, with Yunnan Province and Anhui Province emerging as key production areas. Similar to the Ming Dynasty, abundant Fuling cultivation persisted in Zhejiang and Fujian provinces during the Qing Dynasty. From the Tang and Song dynasties to the Ming and Qing dynasties, the primary production region of Fuling experienced a notable shift, migrating from the north to the south.

In addition to the aforementioned Yunnan, Anhui, Zhejiang, Fujian, and Jiangxi, provinces such as Hubei, Hunan, and Shaanxi also possess extensive records of Fuling distribution. **Figure 3** illustrates the emergence of the Yunnan-Guizhou region and the Dabieshan Mountains as the current primary production areas of Fuling, a trend that began during the Ming Dynasty. Yunnan Province holds 19 distribution records, mainly concentrated in its northern region. It indicates a substantial influence of the LIA on the establishment of the current primary production region for Fuling.

### 4.4 Climate warming may cause Fuling’s original production area to move northward

Since the 1980s, the world’s temperature has predominantly risen (Zhang, 1991). Comparing the distribution areas of Fuling in the Qing Dynasty and modern times, it can be seen that since the Qing Dynasty, the authentic production areas of Fuling have not changed, they are still distributed in Yunnan Province and the Dabieshan Mountains. However, the main producing areas of Fuling have been migrating northwest, and the overall distribution shows a contraction trend. For instance, Fuling is no longer cultivated in Guangdong Province in the south, Zhejiang Province in the southeast, Liaoning Province in the north, and Gansu Province in the northwest. When comparing the modern distribution area of Fuling obtained from the MaxEnt model with the suitable distribution area of Fuling from 2081 to 2100, Guangdong Province in the south is no longer suitable for Fuling distribution, while the suitable distribution area in Henan Province in the north has increased in size. It can be observed that in the next 70 years, the suitable distribution area of Fuling will continue to migrate to the north.

**Figure 7:**
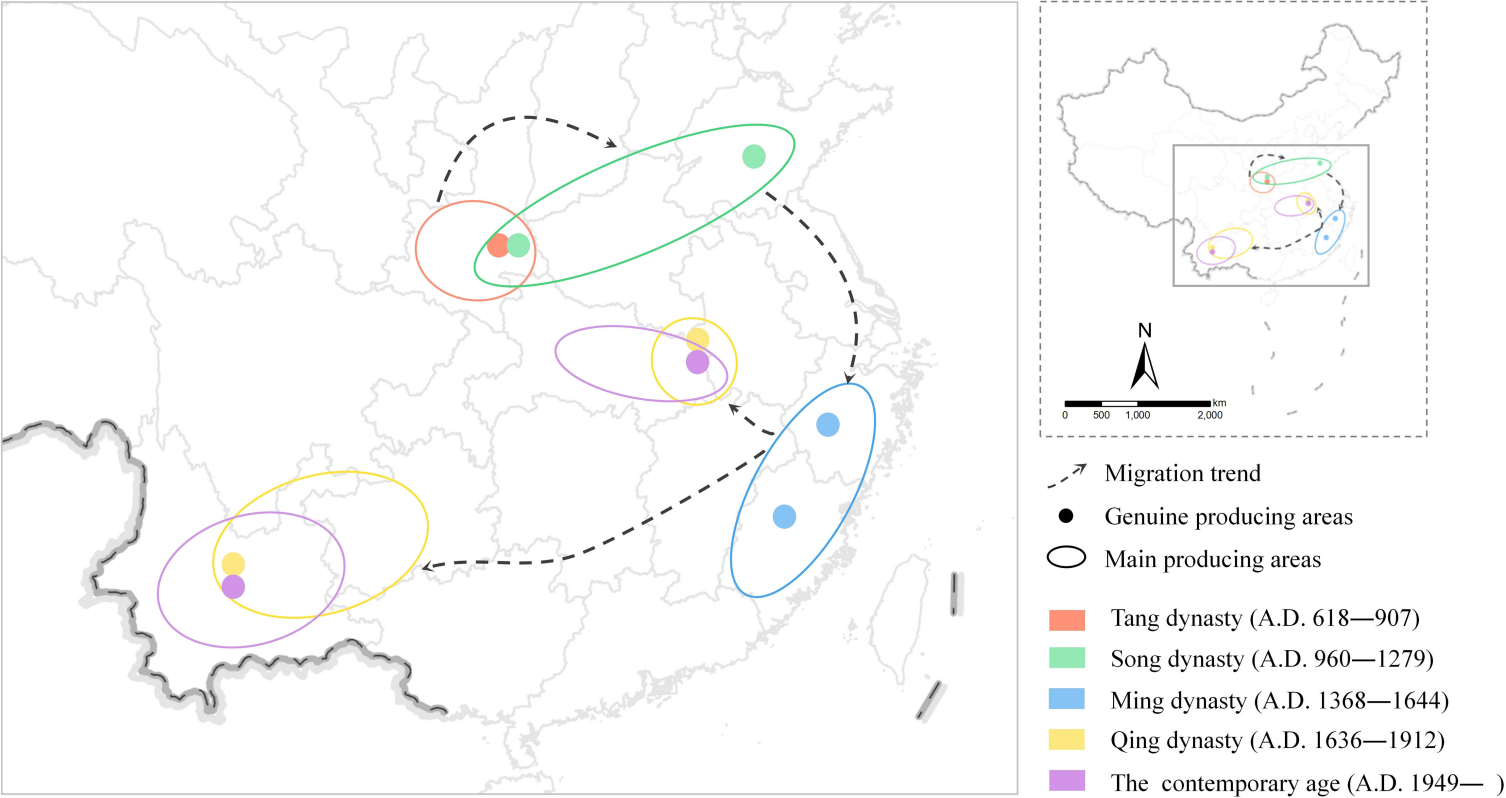
Changes in the distribution of Fuling from the Tang Dynasty to the present day.

The future change trend of Fuling’s primary production region to the north in the context of global warming can be deduced from this study’s results. During the Tang and Song dynasties, Shaanxi Province, situated in the northern region of China, served as the authentic production area for Fuling. While Fuling continues to be cultivated in modern Shaanxi Province, as well as during the Ming and Qing dynasties, it is no longer the primary production area for Fuling. Shandong Province assumed the role of the primary production area during the Song Dynasty, but records of its distribution have declined since the Ming and Qing dynasties. While the MaxEnt model suggests that a section of Shandong Province retains potential as a Fuling distribution area, in reality, there is virtually no evidence of Fuling presence in Shandong Province. Over time, significant climatic changes led Shandong Province to transition from having a substantial Fuling presence to nearly none, while Yunnan Province transformed from lacking Fuling distribution to becoming the primary producing area (**Figure 7**). Beyond climate-related factors, the introduction and cultivation of Fuling through human intervention have also contributed to the long-distance migration of the production area’s focal point. The divergent scenarios observed in these two production regions highlight the impact of both climatic and human factors on the dynamics of medicinal material production areas. This demonstrates that the ideal Fuling distribution areas could potentially revert to Shaanxi and Shandong provinces in northern China if the warming trend continues.

## Data availability statement

The raw data supporting the conclusions of this article will be made available by the authors, without undue reservation.

## Author contributions

YJ: Conceptualization, Data curation, Investigation, Writing – original draft. AR: Data curation, Writing – original draft. XS: Investigation, Writing – original draft. BY: Funding acquisition, Writing – review & editing. HP: Conceptualization, Funding acquisition, Validation, Writing – review & editing. LH: Funding acquisition, Validation, Writing – review & editing.
