# Supplementary material for: Fuling production areas in China: climate and distribution changes (A.D. 618–2100)
Source: Front Plant Sci. 2024 Jan 26;15:1289485. doi: 10.3389/fpls.2024.1289485 (PMC10853409; doi:10.3389/fpls.2024.1289485)
Supplement: Supplementary file 1 [file DataSheet_1.docx]

Supplementary Material

**Fuling Production Areas in China: Climate and Distribution Changes (A.D. 618–2100)**

**Yunlu Jiang^1,2^, Aoyu Ren^1,2^, Xue Sun^1^, Bin Yang^2,3^,Huasheng Peng^1,2,4*^, Luqi Huang^1,2*^**

*** Correspondence:**

Huasheng Peng

hspeng@126.com

Luqi Huang

huangluqi01@126.com


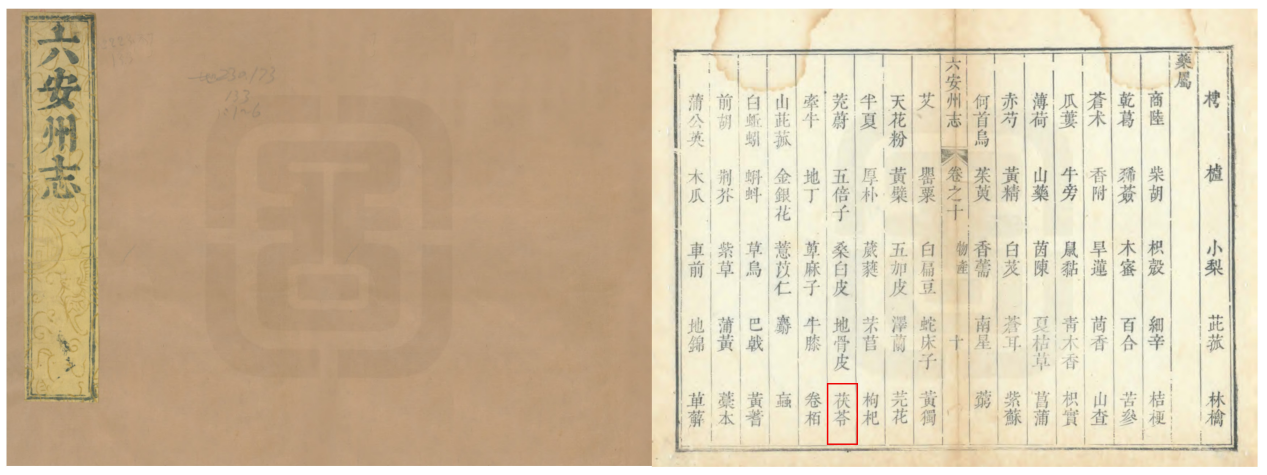


**Supplementary Figure 1** | Records of Fuling(茯苓) in local chronicles *Lu An Zhou Zhi六安州志*. (Image from National Library of China-National Digital Library of China Website (http://read.nlc.cn/user/index)


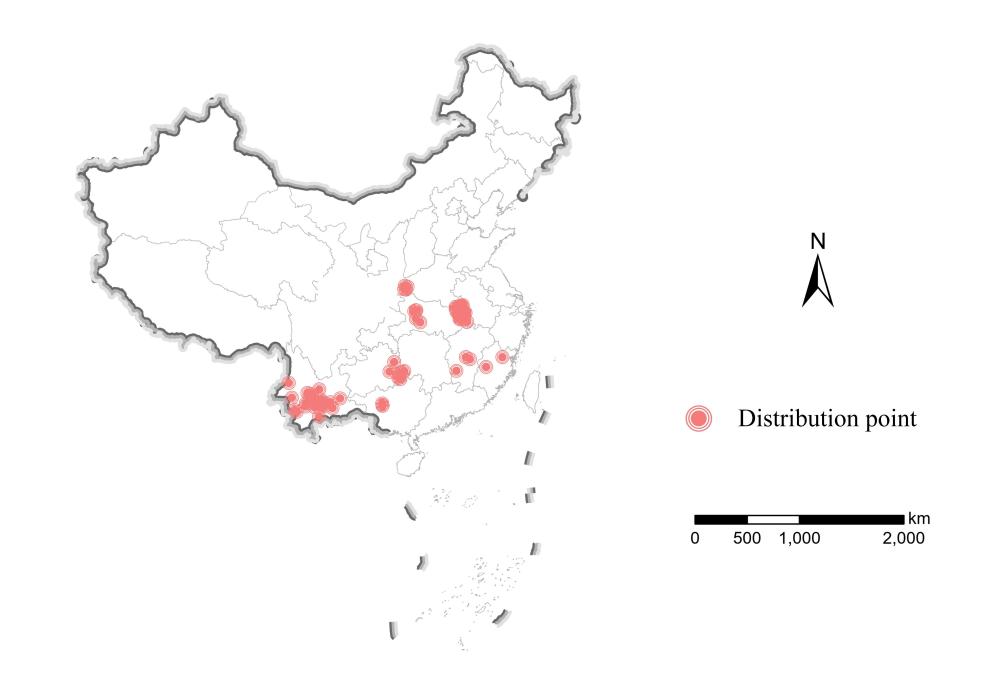


**Supplementary Figure 2** | Occurrence records of modern Fuling.


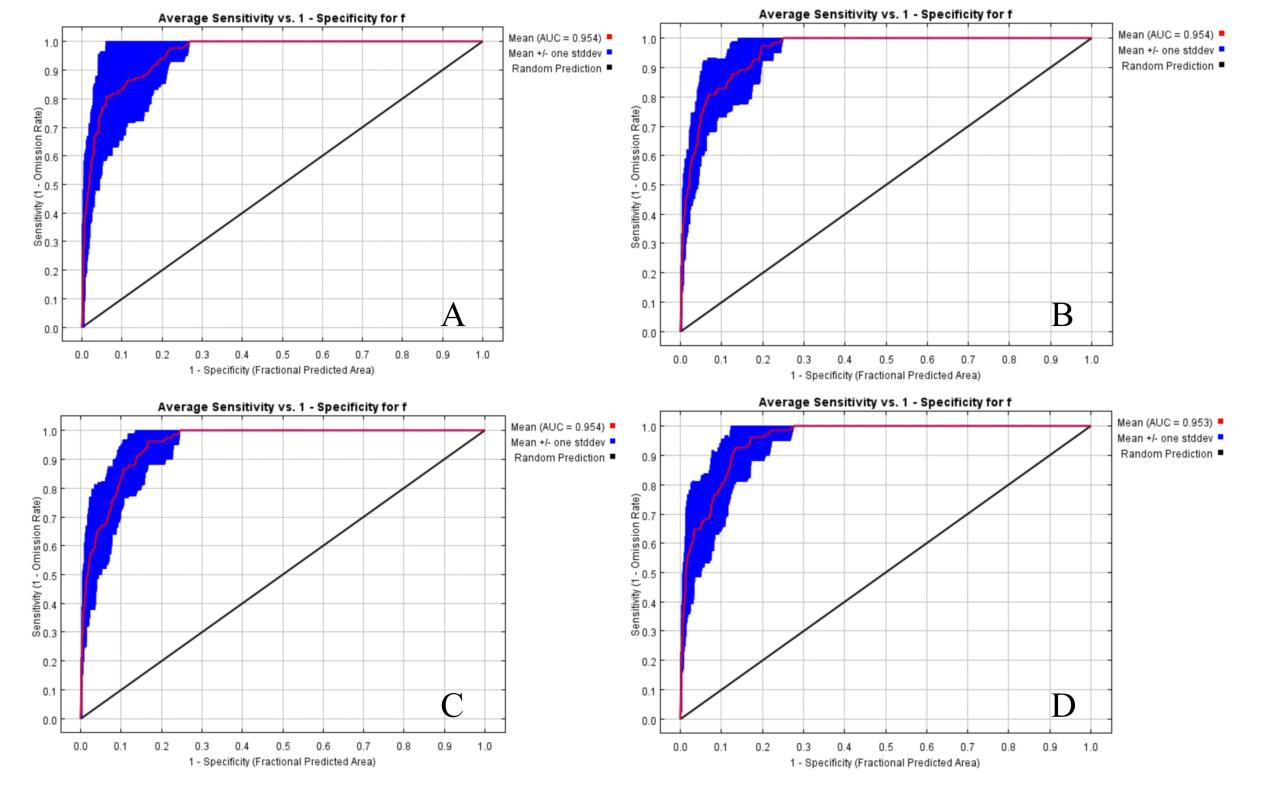


**Supplementary Figure 3** | The ROC curve of Fuling MaxEnt model under different climate scenarios. (A) under the current scenarios; (B) under SSP126 in 2090s; (C) under SSP370 in 2090s; (D) under SSP585 in 2090s.

**Supplementary Table 1 |** English-Chinese comparison of Chinese place names mentioned in this article

| Ancient place names | Chinese name | Modern geographic location |
| --- | --- | --- |
| Anqing | 安庆 | It is located in the southwest of Anhui Province. |
| Chuzhou | 处州 | Lishui City, Zhejiang Province, located in the southwestern part of Zhejiang Province. |
| Fenshui | 分水 | Fenshui Town, Tonglu County, Hangzhou, Zhejiang Province. |
| Fuzhou | 福州 | It is located in the southeastern part of Fujian Province. |
| Guozhou | 虢州 | It is located in present-day Lingbao City, Henan Province and the border area between Henan Province and Shaanxi Province. |
| Huashan | 华山 | It is a mountain located in Huayin City, Weinan City, Shaanxi Province. |
| Huazhou | 华州 | Its jurisdiction corresponds to Huazhou District of Weinan City in present-day Shaanxi Province and the surrounding areas. |
| Lin’an | 临安 | It was located in the Shangcheng and Xiacheng districts of Hangzhou, Zhejiang Province. |
| Ningguo | 宁国 | It is located in the southern part of Xuancheng City, Anhui Province. |
| Songshan | 嵩山 | It is a mountain located in Dengfeng City, Zhengzhou City, Henan Province. |
| Shaoxing | 绍兴 | It is located in the center of Zhejiang Province. |
| Taibaishan | 太白山 | It is located in Shaanxi Province, spanning the counties of Taibai, Meixian, and Zhouzhi. |
| Taishan | 泰山 | It is a mountain located in Tai'an City, Shandong Province. |
| Xijing | 西京 | It was located in present-day Luoyang, Henan Province. |
| Xin’an | 新安 | It was located in present-day Huangshan City, Anhui Province. |
| Xunzhou | 循州 | It was located in present-day Huizhou City, Guangdong Province. |
| Yanzhou | 兖州 | Its jurisdiction was equivalent to Yanzhou City, Tai'an City and Sishui County in present-day Shandong Province. |
| Yanping | 延平 | It is located in Nanping City in the north central part of Fujian Province. |
| Yizhou | 沂州 | Its jurisdiction is equivalent to Linyi, Rizhao and Xintai cities in present-day Shandong Province. |
| Yongzhou | 雍州 | Its jurisdiction corresponds to present-day Xi'an City, Xianyang City and part of Tongchuan City in Shaanxi Province. |
| Zhongnanshan | 终南山 | It is located in the middle of the Qinling Mountains in Shaanxi Province, south of Xi'an. |
